# Clinical Validation of Therapeutic Drug Monitoring of Imipenem in Spent Effluent in Critically Ill Patients Receiving Continuous Renal Replacement Therapy: A Pilot Study

**DOI:** 10.1371/journal.pone.0153927

**Published:** 2016-04-19

**Authors:** Aiping Wen, Zhe Li, Junxian Yu, Ren Li, Sheng Cheng, Meili Duan, Jing Bai

**Affiliations:** 1 Department of Pharmacy, Beijing Friendship Hospital, Capital Medical University, Beijing, China; 2 School of Chemical Biology and Pharmaceutical Sciences, Capital Medical University, Beijing, China; 3 Department of Critical Care Medicine, Beijing Friendship Hospital, Capital Medical University, Beijing, China; Johannes Kepler University Linz, AUSTRIA

## Abstract

**Objectives:**

The primary objective of this pilot study was to investigate whether the therapeutic drug monitoring of imipenem could be performed with spent effluent instead of blood sampling collected from critically ill patients under continuous renal replacement therapy.

**Methods:**

A prospective open-label study was conducted in a real clinical setting. Both blood and effluent samples were collected pairwise before imipenem administration and 0.5, 1, 1.5, 2, 3, 4, 6, and 8 h after imipenem administration. Plasma and effluent imipenem concentrations were determined by reversed-phase high-performance liquid chromatography with ultraviolet detection. Pharmacokinetic and pharmacodynamic parameters of blood and effluent samples were calculated.

**Results:**

Eighty-three paired plasma and effluent samples were obtained from 10 patients. The Pearson correlation coefficient of the imipenem concentrations in plasma and effluent was 0.950 (P<0.0001). The average plasma-to-effluent imipenem concentration ratio was 1.044 (95% confidence interval, 0.975 to 1.114) with Bland-Altman analysis. No statistically significant difference was found in the pharmacokinetic and pharmacodynamic parameters tested in paired plasma and effluent samples with Wilcoxon test.

**Conclusion:**

Spent effluent of continuous renal replacement therapy could be used for therapeutic drug monitoring of imipenem instead of blood sampling in critically ill patients.

## Introduction

Serious infections, including sepsis, remain major cause of morbidity and mortality in critically ill patients [1–6]. Imipenem has been extensively used to treat serious infections when the main suspected pathogens are Gram-negative bacteria, due to its broad spectrum of coverage and highly potent therapeutic effects [7, 8]. It has been highlighted that early, appropriately dosed antibiotics is beneficial for critically ill patients [9–13]. However, appropriate antibiotic dosing in critically ill patients is still challenging, because of the rapidly dynamic physiology, decreasing levels of susceptibility of bacteria and unpredictable pharmacokinetic characteristics [14–16]. In addition, there are indications of high incidence of suboptimal antibiotic concentrations and therapeutic failure of β-lactams in critically ill patients, due to the wide pharmacokinetic variability of imipenem [13, 15, 17, 18]. As a β-lactam, therapeutic drug monitoring (TDM) of imipenem has not been widely investigated as a routine intervention because of the wide therapeutic window [19, 20]. Nevertheless, it has been illustrated that TDM is associated with optimal β-lactam concentrations and improved clinical outcomes in critically ill patients [20–22].

Continuous renal replacement therapy (CRRT), particularly continuous venovenous haemofiltration (CVVH) and continuous venovenous haemodiafiltration (CVVHDF), are increasingly used in the routine clinical management of critically ill patients [23, 24]. CRRT plays a significant role in critically ill patients with acute kidney injury (AKI), and has also been used for the treatment of some non-renal indications, such as severe sepsis [23–26]. The use of CRRT makes the design of the optimal dosage regimen for the critically ill patients more complicated. Patients receiving CRRT might be underdosed with published dosing recommendations due to variability, and currently used CRRT may be more efficient than that reported by the literatures [27]. Moreover, lower than anticipated or desired systemic antimicrobial exposure, therapeutic failure, and the emergence of breakthrough resistance are indicated for critically ill patients receiving CRRT [28–31]. Hence, TDM of β-lactams is suggested as a desirable intervention for this population [32]. However, blood sampling is commonly necessary for traditional TDM, and the problem of diagnostic blood draws in critically ill patients has been addressed, which may contribute to anemia and be associated with morbidity [33]. Moreover, antibiotic concentrations are usually measured by high-pressure liquid chromatography (HPLC), and blood sample preparation can lead to long latency time and low throughput for TDM [34].

Previous study has showed that there is a strong correlation between plasma free and dialysate effluent piperacillin concentrations in patients receiving continuous venovenous hemodialysis (CVVHD) [34], which suggests that an equilibrium may be achieved between plasma and dialysate. If this inference is justified, spent effluent of CVVHD may be a noninvasive alternative to blood sampling for TDM of small molecules. Moreover, effluent sample is ready for analysis without further preparation, which simplifies sample preparation and promotes the productivity of TDM. However, it is unclear whether this could be extrapolated to other drugs and other types of CRRT in critically ill patients. The primary objective of this study was to develop and clinically validate a method of analyzing imipenem in spent effluent in critically ill patients with CRRT. The present study also aimed to conduct preliminary evaluation of the pharmacokinetic and pharmacodynamic characteristics of imipenem in these patients.

## Materials and Methods

### Study Design

This prospective open-label study was conducted in the intensive care unit (ICU) of Beijing Friendship Hospital, Beijing, China. Patients were eligible for inclusion if the following criteria were met: 1) adult; 2) admission to ICU; 3) treatment with imipenem and CRRT simultaneously. Patients who were judged inappropriate to get frequent blood samples by the attending physician were excluded from this study. The study protocol was approved by the Research Ethics Committee of Beijing Friendship Hospital. All of the eligible patients or their legal guardians were informed the essentials of this study, and written informed consent was obtained before enrolling each patient into this study.

### Medications

Patients enrolled into this study received imipenem-cilastatin (500mg/500mg, Merck Sharp & Dohme Corp., USA) as part of their medical care. Dosage regimen was determined by the attending physicians according to clinical indication and institutional dosing guidelines. Dosages represented by the quantity of imipenem of 500 mg every 6 h, 500 mg every 8 h and 1 g every 8 h were commonly prescribed. Both of the doses of 500mg and 1 g were suspended and transferred to 100 ml of an appropriate infusion solution, and administered by intravenous infusion pump over 1 h. Detailed dosage regimen and specific administration time were recorded for each enrolled patient.

### CRRT Procedures

All patients were treated with CRRT using the Gambro PrismaFlex system with Gambro Prismaflex M100 AN-69 hemofilter sets. Vascular access was obtained by introduction of an ARROW Two-Lumen Hemodialysis Catheterization Set (12Fr, 20cm, Arrow International Inc., USA) into the jugular vein or an ARROW Large-Bore Multi-lumen Central Venous Catheterization Set (12Fr, 16cm, Arrow International Inc., USA) into the femoral vein. The type and dosing of CRRT were managed by the attending physicians. The parameters such as blood flow rate, dialysate rate and ultrafiltration rate were set and adjusted by therapeutic needs. Replacement fluids were delivered by a combination of pre and post filter. The extracorporeal circuit was anticoagulated with sodiumcitrate. The uptime of the hemofilter sets was generally 24–48 h. The types and parameters of CRRT were recorded for each patient in detail. Urine output was obtained from the nursing records.

### Sample Collection and Storage

In order to obtain plasma imipenem concentrations at or near steady state, sampling was performed at least 24 h after initiation of the CRRT and imipenem therapy [35]. Blood and effluent samples were collected pairwise before and 0.5, 1, 1.5, 2, 3, 4, 6, and 8 h (for the dose of 1 g every 8 h) after imipenem administration. Blood samples (0.6 mL) were drawn from the red access port of the central line for CRRT, and collected into tubes containing heparin as anticoagulant. Effluent samples (approximately 1.2 mL) were collected from the CRRT apparatus directly into polypropylene tubes. All of the samples were placed in ice box immediately, and processed within 2 h. Because imipenem is rapidly hydrolyzed in plasma through a pH-dependent reaction [36], morpholinopropanesulfonic acid (MOPS, ultra pure grade; Amresco, USA) buffer (0.126 M, pH 6.8) was served as stabilizing solution. Blood samples were centrifuged (3,000×g, 10 min) and aliquots of plasma were mixed 1:1 with stabilizing solution [37]. Effluent samples were directly mixed 1:1 with stabilizing solution. Stabilized plasma and effluent samples were stored at -70°C until analysis. If CRRT was interrupted, sampling was halted, and resampling was allowed after CRRT was reestablished. Complete profiles of medical history, physical examination and laboratory tests were obtained and reviewed prior to collection of samples.

### Sample Analysis and Validation

Analysis of imipenem concentrations in plasma and effluent were based on previously validated high performance liquid chromatography—ultraviolet detection (HPLC-UV) methods [37–39] with a few modifications. Chromatography was performed on a ZORBAX SB-C8 column (5μm, 4.6 x 250 mm; Agilent, USA), maintained at 30°C. The ultraviolet detector was set at 298 nm. A gradient elution of ammonium acetate buffer (0.5 M, pH 6.8) and acetonitrile was used as the mobile phase with a flow rate of 1 mL/min. Imipenem monohydrate (I1K226, 0.932 mg/mg; USP) was used for the preparation of standard solutions, and ceftazidime was used as the internal standard.

Two hundred microliters of stabilized plasma and effluent samples were mixed with 20 μL of 500 μg/mL ceftazidime, respectively. The effluent samples were injected directly into the HPLC system. The plasma samples were deproteinized using Amicon Ultra-0.5 centrifugal filters (3 kDa, Millipore, USA), and the filtrates were injected into the HPLC system.

The lower limit of quantification (LOQ) was 0.3 μg/mL for both of the plasma and effluent samples. The linearity of the standard curve was assessed with 1/x^2^ weighting over a concentration range of 0.3 to 200.0 μg/mL. Accuracy and precision were evaluated with quality control samples at concentrations of 0.5, 2.0, 30.0, and 75.0 μg/mL in triplicate. Stability was assessed by storing stabilized quality control samples (2.0, 30.0, and 75.0 μg/mL) at −70°C and 20°C for 30 days and 6 h, respectively. The stability of patients’ samples stored in ice box (2–8°C) for 2 h was also evaluated.

### Pharmacokinetic and Pharmacodynamic Analysis

Pharmacokinetic analysis was performed by Drug and Statistics (DAS, version 2.0, Mathematical Pharmacology Professional Committee of China, Shanghai, China) using non-compartment model [40]. As a β-lactam, the pharmacodynamic predictor of clinical efficacy and risk of developing microbial resistance to imipenem is commonly indicated by the percentage of free drug concentrations remain above the minimum inhibitory concentration (MIC) of the pathogen (*f* T > MIC), and a target of at least 40% was recommended [7, 41–43]. For critically ill patients, the pharmacodynamic predictor of the β-lactams was not clearly defined. It was indicated that *f* T > 4–5 × MIC could maximize the likelihood of clinical cure in patients with severe infections [19, 20], and the target of at least of 60% is suggested for bolus infusion [44]. Therefore, both of *f* T>MIC and *f* T > 4 × MIC were calculated according to the methods of Fish, *et al* [35]. For the patient without an identified pathogen or without the MIC for imipenem, the pharmacodynamic predictor was not calculated.

### Statistical Analysis

In the process of analytical method validation, bias was defined as the difference between the analytical concentrations and the nominal concentrations, expressed as a percentage. The effluent analysis was clinically validated by comparing the imipenem concentrations in paired plasma and effluent samples from patients by Passing-Bablok regression and Bland-Altman analysis using MedCalc, version 11.4.2 (MedCalc Software, Ostend, Belgium). Furthermore, the Pearson correlation was calculated to determine the correlation between the concentrations in plasma and effluent samples. The Wilcoxon test for paired samples was applied to the comparisons of pharmacokinetic and pharmacodynamic parameters. A *P* value of <0.05 was considered statistically significant.

## Results

### Subject Characteristics

A total of 10 patients were enrolled in this study and completed the scheduled sampling. The median age and weight of the patients were 64 years (range, 32 to 87 years) and 70 kg (range, 45–90 kg), and 8 patients (80.0%) were male. The characteristics of the patients are summarized in Table 1. Eight of the patients were diagnosed with severe sepsis, and the other two patients were diagnosed with serious abdominal infection and severe acute pancreatitis, respectively. Eight of the patients were diagnosed with acute kidney injury. Half of the patients were with hypoalbuminaemia. The isolated pathogens were *Enterobacter aerogenes* in 2 patients, *Acinetobacter baumannii* in 2 patients (20.0%), *Escherichia coli* in 2 patients (20.0%), *Klebsiella pneumoniae* in 1 patient (10.0%), *Pseudomonas aeruginosa* in 1 patient (10.0%), and not specified in 3 patients (30.0%). The dosages of imipenem were 500 mg every 6 h in 7 patients (70.0%), 1 g every 8 h in 2 patients (20.0%) and 0.5 g every 8 h in 1 patient (10.0%).

**Table 1:** Characteristics of the patients.

Patient	Age (yr)	Sex ^a^	Wt (kg)	APACHE II Score ^b^	Principal diagnosis	Isolated pathogen (imipenem MIC in μg/mL)	Imipenem dosing	Outcome
1	33	M	80	15	Pneumonia, severe sepsis, hypoproteinemia	*Enterobacter aerogenes* (1)	0.5g q6h	Died
2	59	M	80	17	Abdominal infection, severe sepsis, shock	*Klebsiella pneumoniae* (1)	0.5g q6h	Survived
3	69	M	70	13	Pneumonia, acute kidney injury, severe sepsis, myocardial injury, acute liver injury, hypoproteinemia	None	0.5g q6h	Survived
4	74	F	45	20	Pneumonia, severe sepsis, acute kidney injury, acute liver injury, shock, acute myocardial ischemia, hypoproteinemia	*Acinetobacter baumannii* (1)	0.5g q6h	Died
5	47	M	90	16	Abdominal infection, acute kidney injury, acute liver injury	*Escherichia coli* (1)	1g q8h	Survived
6	32	M	70	20	Severe acute pancreatitis, acute kidney injury, acute liver injury, myocardial injury, hypoproteinemia	None	1g q8h	Died
7	87	M	70	23	Pneumonia, severe sepsis, shock, acute liver injury, acute kidney injury	*Escherichia coli* (16)	0.5g q6h	Died
8	50	M	70	26	Pneumonia, severe sepsis, shock, acute liver injury, chronic renal insufficiency accompanied with acute kidney injury, acute left heart failure	None	0.5g q6h	Died
9	76	F	75	26	Multiple organ dysfunction syndrome, acute left heart failure, acute liver injury, acute kidney injury, pneumonia, severe sepsis, shock	*Pseudomonas aeruginosa* (1)	0.5g q6h	Survived
10	78	M	60	15	Pneumonia, severe sepsis, shock, chronic renal insufficiency accompanied with acute kidney injury, hepatic dysfunction, hypoproteinemia	*Acinetobacter baumannii* (16)	0.5g q8h	Died

APACHE, Acute Physiology and Chronic Health Evaluation; MIC, minimum inhibitory concentration.

^a^ F, female; M, male.

^b^ During or near the day of sampling.

Urine output and details of CRRT of the patients were illustrated in Table 2. Eight patients received CRRT because of AKI, two for severe sepsis. Nine patients were treated with CVVHDF, and one with CVVH.

**Table 2:** Urine output and details of continuous renal replacement therapy.

Patient	Urine output ^a^ (mL/24 h)	CRRT Type	Blood flow rate ^a^ (mL/min)	Dialysate rate ^b^ (mL/h)	Ultrafiltration rate ^b^ (mL/h)	Duration of filter (h)
1	1940	CVVHDF	150	1000	1380	56
2	2125	CVVHDF	150	1000	1800	61
3	1220	CVVH	150	-	2520	8
4	260	CVVHDF	150	1000	1310	68
5	40	CVVHDF	150	500	1950	37
6	55	CVVHDF	150	1000	1440	31
7	65	CVVHDF	150	1500	920	20
8	0	CVVHDF	150	1000	1400	11
9	500	CVVHDF	150	1000	1180	2
10	30	CVVHDF	150	1000	1500	47

CRRT, continuous renal replacement therapy; CVVHDF, continuous venovenous hemodiafiltration; CVVH, continuous venovenous hemofiltration.

^a^ During the day of sampling.

^b^ Documented at the initiation of sampling, could be adjusted based on the patient’s condition.

### Validation of Analytical Methods

The plasma and effluent analytical method showed a good linearity over the imipenem concentration range. Correlation coefficients were within the range of 0.9990 to 1.0000 for the entire process of analysis. The mean measured concentrations were between 93.96% and 105.25% of the nominal concentration for plasma and between 97.24% and 106.48% for effluent. The within-day variations were between 1.04% and 3.03% for plasma and between 0.31% and 1.73% for effluent, and the day-to-day variations were between 0.63% and 5.11% for plasma and between 0.63% and 1.13% for effluent. Both stabilized plasma and effluent samples were stable at −70°C and 20°C for 30 days and 6 h, respectively. The patients’ samples were stable in ice box for 2 h. All variations were well within the desired limits of 15%.

### Clinical Validation

**Fig 1:**
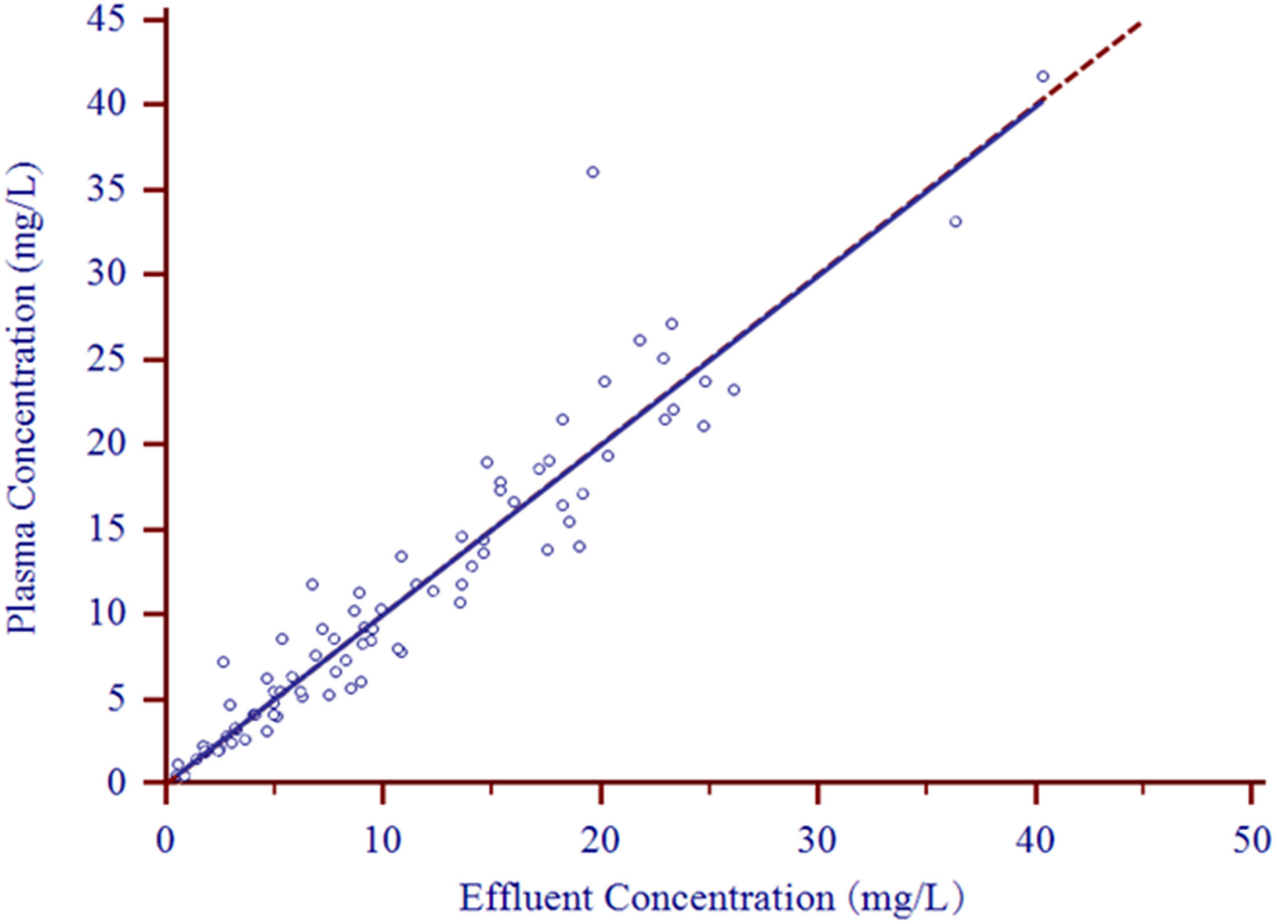
Scatter plot with Passing-Bablok fit of plasma and effluent concentrations in mg/liter (n = 83). Identity lines are presented as dashed lines, and regression lines are depicted as solid lines. The regression line of the imipenem plasma/effluent concentration ratio has a slope of 0.997 (95% CI, 0.935 to 1.078) and an intercept of -0.039 (95% CI, -0.514 to 0.247).

**Fig 2:**
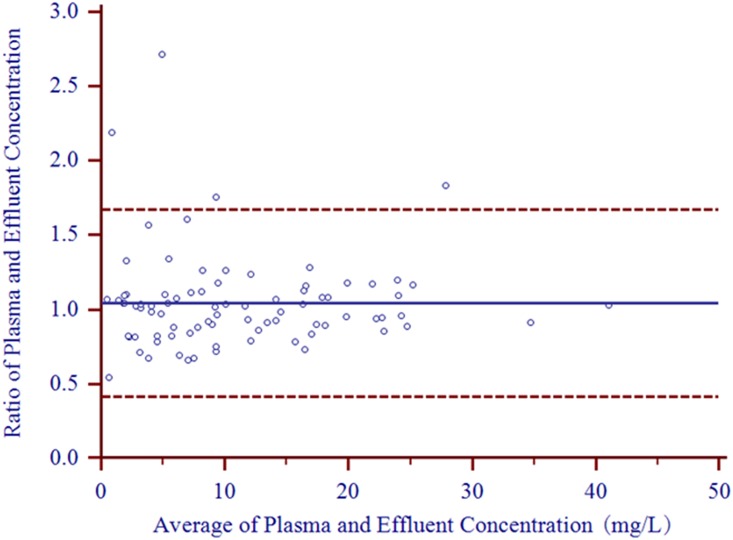
Bland-Altman plot of plasma/effluent concentration ratios compared to average plasma and effluent concentrations (n = 83). The line representing the bias is presented as a solid line, and the 95% limits of agreement are presented as dashed lines. The bias is 1.044 (95% CI, 0.975 to 1.114), and the lower and upper 95% limits of agreement are 0.417 (95% CI, 0.298 to 0.537) and 1.671 (95% CI, 1.552 to 1.791).

Eighty-three paired plasma and effluent samples from 10 patients were included in the clinical validation of the effluent analysis. The Pearson correlation showed a correlation coefficient of 0.950 (*P*<0.0001) for the imipenem concentrations in plasma and effluent. Passing-Bablok regression between the plasma and effluent imipenem concentrations showed a proportional bias of 0.997 (95% confidence interval [CI], 0.935 to 1.078) and a constant bias of -0.039 (95% CI, -0.514 to 0.247) (Fig 1). A linear relationship between plasma and effluent imipenem concentrations was indicated by the Cusum linearity test (P>0.10). Bland-Altman assessment showed a good agreement between analyses of imipenem concentrations in plasma and effluent, with 4.8% (4/83) of observations for imipenem falling outside 95% limits of agreement (Fig 2). The observed bias for the mean plasma-to-effluent imipenem concentration ratio versus the mean concentration in plasma and effluent was 1.044 (95% CI, 0.975 to 1.114, Fig 2).

### Pharmacokinetic and Pharmacodynamic Evaluation

**Fig 3:**
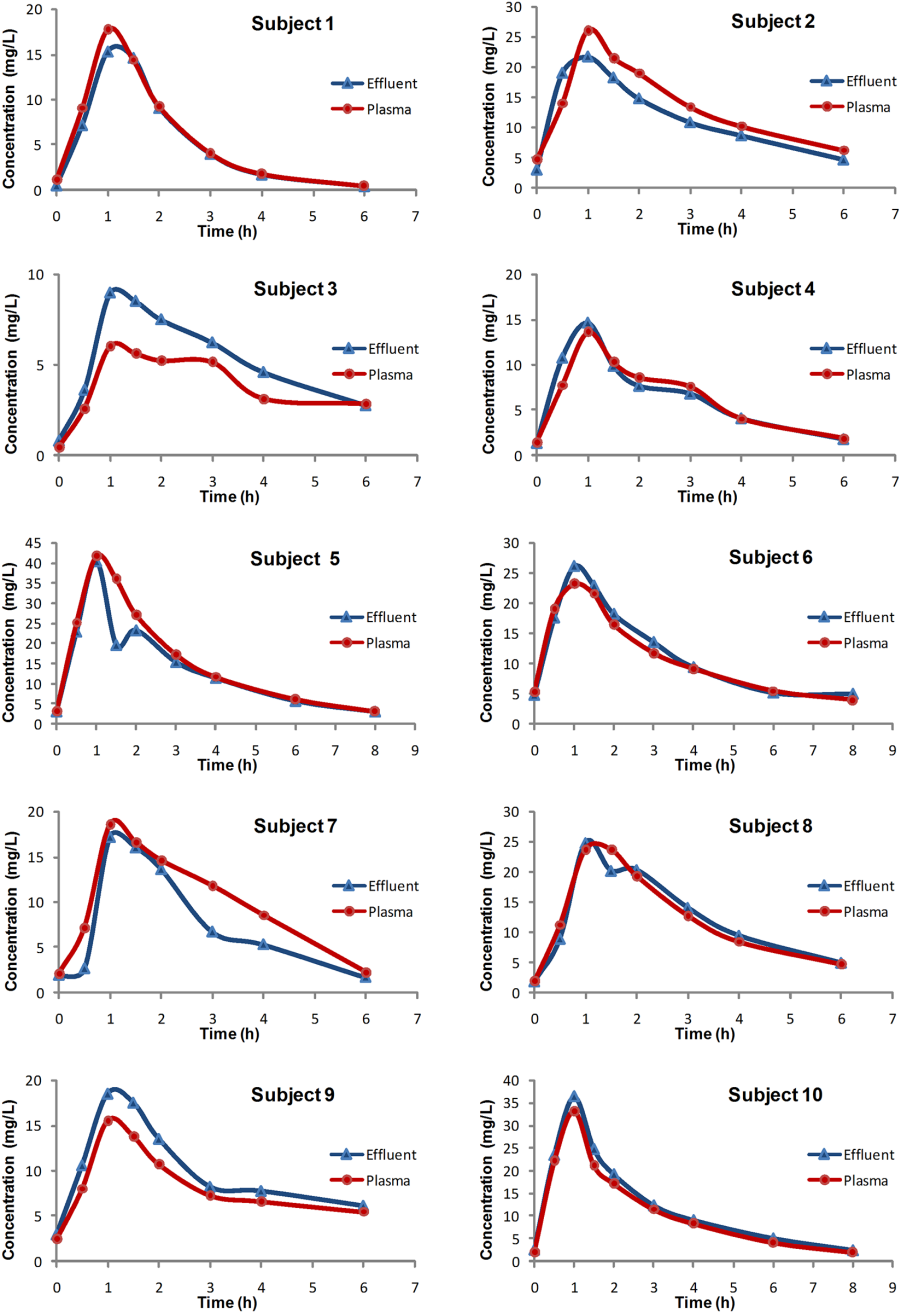
Plasma and effluent concentrations of imipenem during continuous renal replacement therapy for 10 patients. The X axis represents postinfusion times.

Plasma and effluent concentration-versus-time profiles for imipenem during CRRT for each patient are shown in Fig 3. Pharmacokinetic parameters of imipenem in plasma and effluent are displayed in Table 3. The Wilcoxon test for paired samples showed no statistically significant difference between medians of all pharmacokinetic parameters in plasma and effluent samples.

**Table 3:** Pharmacokinetic parameters of imipenem in plasma and effluent samples.

Patient	Imipenem dosing	CRRT Type	Cmax (μg /mL)		Cmin (μg /mL)		AUC_0-t_ (μg·h/mL)		t_1/2_ (h)		CL (mL/min/kg)		Vd (L/kg)	
			Plasma	Effluent	Plasma	Effluent	Plasma	Effluent	Plasma	Effluent	Plasma	Effluent	Plasma	Effluent
1	0.5g q6h	CVVHDF	17.79	15.34	0.50	0.47	35.27	32.72	0.86	0.84	2.92	3.14	0.22	0.23
2	0.5g q6h	CVVHDF	26.11	21.75	4.66	2.97	81.11	69.76	2.50	2.28	1.01	1.22	0.22	0.24
4	0.5g q6h	CVVHDF	13.61	14.64	1.47	1.39	38.25	38.62	1.84	1.88	4.28	4.25	0.68	0.69
7	0.5g q6h	CVVHDF	18.58	17.16	2.12	1.71	59.56	45.01	2.60	1.37	1.52	2.46	0.34	0.29
8	0.5g q6h	CVVHDF	23.75	24.81	1.96	1.79	74.70	75.74	1.69	1.96	1.42	1.33	0.21	0.23
9	0.5g q6h	CVVHDF	15.49	18.51	2.47	3.02	49.88	60.42	7.20	2.24	1.04	1.50	0.65	0.29
Median (range)			18.19 (13.61–26.11)	17.84 (14.64–24.81)	2.04 (0.50–4.66)	1.75 (0.47–3.02)	54.72 (35.27–81.11)	52.72 (32.72–75.74)	2.17 (0.86–7.20)	1.92 (0.84–2.28)	1.47 (1.01–4.28)	1.98 (1.22–4.25)	0.28 (0.21–0.68)	0.27 (0.23–0.69)
5	1g q8h	CVVHDF	41.66	40.34	3.24	3.16	123.40	107.16	2.07	2.00	1.39	1.60	0.25	0.28
6	1g q8h	CVVHDF	23.23	26.15	4.00	4.91	86.09	91.77	2.72	2.22	2.40	2.36	0.57	0.45
Median (range)			32.45 (23.23–41.66)	33.25 (26.15–40.34)	3.62 (3.24–4.00)	4.04 (3.16–4.91)	104.75 (86.09–123.40)	99.47 (91.77–107.16)	2.40 (2.07–2.72)	2.11 (2.00–2.22)	1.90 (1.39–2.40)	1.98 (1.60–2.36)	0.41 (0.25–0.571)	0.37 (0.28–0.45)
3	0.5g q6h	CVVH	6.02	8.95	0.46	0.84	23.79	32.27	4.57	2.79	2.80	2.74	1.11	0.66
10	0.5g q8h	CVVHDF	33.11	36.34	1.95	2.37	85.41	95.31	1.91	2.31	1.53	1.33	0.25	0.27
P value ^a^			0.625		0.922		0.846		0.160		0.232		0.557	

CRRT, continuous renal replacement therapy; CVVHDF, continuous venovenous hemodiafiltration; CVVH, continuous venovenous hemofiltration.

^a^ *P* values were calculated by the Wilcoxon test.

Pharmacodynamic parameters of imipenem for the patients are displayed in Table 4. No statistically significant difference was found between medians of *f* T>MIC or *f* T>4 × MIC in plasma and effluent samples, respectively. Both of *f* T>MIC in plasma and effluent samples showed that the pathogens with a MIC of 1 μg/mL would be adequately treated with the doses of 0.5 g every 6 h and 1 g every 8 h. However, when *f* T>4 × MIC was used, only 2 patients (50.0%) with the doses of 0.5 g every 6 h and the patient with the dose of 1 g every 8 h were adequately treated. It was also found that these were the only three patients survived, the other two patients predicted undertreated by *f* T>4 × MIC both passed away. For the pathogens with a MIC of 16 μg/mL, neither of the two patients (with the doses of 0.5 g every 6 h and 1 g every 8 h, respectively) was adequately treated based on the values of both *f* T>MIC and *f* T>4 × MIC, and both passed away.

**Table 4:** Pharmacodynamic parameters of imipenem in plasma and effluent samples.

Patient	Imipenem dosing	CRRT Type	MIC (μg /mL)	%*f* T>MIC		%*f* T>4×MIC		Outcome
				Plasma	Effluent	Plasma	Effluent	
1	0.5g q6h	CVVHDF	1	59.32^#^	55.32^#^	30.75	27.24	Died
2	0.5g q6h	CVVHDF	1	196.29^#^	168.55^#^	112.88^#^	92.68^#^	Survived
4	0.5g q6h	CVVHDF	1	115.41^#^	121.24^#^	54.13	58.62	Died
9	0.5g q6h	CVVHDF	1	474.38^#^	157.24^#^	234.38^#^	82.55^#^	Survived
Median (range)				155.85 (59.32–474.38)	139.24 (55.32–168.55)	83.51 (30.75–234.38)	70.59 (27.24–92.68)	
5	1g q8h	CVVHDF	1	139.47^#^	133.52^#^	87.63^#^	83.46^#^	Survived
7	0.5g q6h	CVVHDF	16	9.35	2.31	-77.39	-43.44	Died
10	0.5g q8h	CVVHDF	16	25.11	34.17	-22.76	-23.58	Died
P value ^a^				0.297		0.578		

CRRT, continuous renal replacement therapy; CVVHDF, continuous venovenous hemodiafiltration; CVVH, continuous venovenous hemofiltration; MIC, minimum inhibitory concentration.

^a^ *P* values were calculated by the Wilcoxon test.

^#^Target was achieved.

## Discussion

To our knowledge, this is the first study to investigate the possibility of using spent effluent of CRRT to perform TDM of imipenem for critically ill patients instead of blood samples. We found the imipenem concentration in effluent samples was in good agreement with the plasma imipenem concentration. The mean ratio of effluent and plasma imipenem concentrations was approximately 1. It has been shown that the drug concentration in ultrafiltrate or dialysate divided by that in plasma was mainly influenced by plasma protein binding of the drugs [24, 45, 46]. Therefore, this result may be explained by the low plasma protein binding of imipenem, which is showed as 9% in healthy volunteers [47]. Hypoproteinemia is very common in critically ill patients, and even lower protein binding of imipenem may be expected [24, 48]. Previous study illustrates a strong correlation between plasma free and dialysate piperacillin concentrations in patients receiving CVVHD [34]. Protein binding of piperacillin is about 20–30% in healthy volunteers and patient populations [49]. Taking these into account, it may be possible to use the effluent of CRRT to perform TDM of other drugs with low or moderate plasma protein binding, especially for critically ill patients.

Moreover, though the mean ratio of effluent and plasma imipenem concentrations was approximately 1, the limits of agreement of the ratio were between 0.417 and 1.671, which had a discrepancy of approximately 3-fold, and a larger bias was seen in the plasma-to-effluent concentration ratio in the low-concentration area. The bias may be mainly explained by the factors of potential adsorption by filter membrane and the disparity of filter duration for each patient [45, 50, 51]. It is indicated that a proteinaceous secondary membrane is formed when blood exposes to the filter, possibly as soon as the first few minutes of CRRT [52]. And the formation of the proteinaceous membrane over the filter may hinder convective solute removal and reduce transmembrane clearance as the proteinaceous membrane thickens [51]. The time limits for the filters are typically set at 24–48 h. However, we may extend the duration of filters up to 96 h in the spirit of cost containment for some patients, unless of clotting. Limited importance is attached to the issue of filter performance over time, because of the difficulty to assess. The ratio of effluent and plasma urea nitrogen is commonly recommended as a surrogate marker of filter performance [53]. However, effluent creatinine/serum creatinine is suggested to be a better marker than the ratio of urea nitrogen in a recent study, because of a better sensitivity for filter performance [51]. It is a pity that we did not evaluate the performance of the filter in this study. This will be included in our further studies to verify that effluent is well equilibrated with plasma.

In this study, we found no statistically significant difference between medians of all pharmacokinetic and pharmacodynamic parameters in plasma and effluent samples, which further validated the rationality of using effluent samples to perform imipenem analysis instead of blood samples. However, it should be noted that pharmacodynamic parameters was significantly underestimated by effluent sampling (approximately three times) in one patient (subject 9). The reason for this is not clear, and further investigation is needed. Besides that, we found *f* T>4×MIC may be a better indicator of clinical efficacy for imipenem in critically ill patients. For the pathogens with MIC = 16μg/mL, the doses of 0.5 g every 6 h and 1 g every 8 h could not achieve the therapeutic target and may cause therapeutic failure. Because of the limited samples in this study, these findings need to be justified by further investigations.

There are a few limitations to be taken in consideration in this study. Limited number of patients and only the CRRT types of CVVHDF and CVVH were included. Furthermore, though we tried to limit the influential factors as minimal as possible, the factors, such as the patient's condition and the duration of the hemofilter were not well controlled, this may cause the bias.

In conclusion, a method for analyzing imipenem in spent effluent in critically ill patients with CRRT is developed and clinically validated in this pilot study conducted in a real-world clinical setting. It suggests that reliable measurement of drug concentrations in spent effluent of CRRT may facilitate therapeutic drug monitoring of imipenem in this population. Further investigation is needed to justify the clinical value of our results.
